# Foamy Macrophages and Blue Histiocytes as Diagnostic Clues to Acid Sphingomyelinase Deficiency

**DOI:** 10.1002/jha2.70098

**Published:** 2025-08-27

**Authors:** Andrea Franch, Mònica Ribell Bachs, Jose Ramón Álamo Moreno

**Affiliations:** ^1^ Department of Haematology Hospital Clinic de Barcelona Barcelona Spain; ^2^ Department of Internal Medicine Hospital General de Granollers Granollers Spain; ^3^ Haematopathology Unit, Department of Pathology Hospital Clinic de Barcelona Barcelona Spain

**Keywords:** acid sphingomyelinase deficiency, blue histiocytes, bone marrow morph, bone marrow pathology, cytopenia, lysosome storage disorders

1

We report the case of a 54‐year‐old Spanish male, born to consanguineous parents, with no relevant medical history, who presented with progressive interstitial lung disease, hepatosplenomegaly with portal hypertension, and pruritic cutaneous lesions. Laboratory tests revealed microcytic anaemia (haemoglobin 89 g/L, MCV 79 fL) and thrombocytopenia (platelets 93 ×10⁹/L), with a normal white blood cell count. Viral hepatitis and full autoimmune screenings were negative except for an elevated angiotensin‐converting enzyme (ACE), low‐titre positive anti‐nuclear antibodies (ANA), and polyclonal hypergammaglobulinaemia. Whole‐body CT scan showed splenic lesions and paravertebral masses.

Differential diagnoses included systemic autoimmune conditions, connective tissue disorders, sarcoidosis, infections (e.g., *Leishmania*, tuberculosis), and haematological malignancies, such as lymphoproliferative or myeloproliferative disorders.

The peripheral blood smear showed no abnormalities. Bone marrow aspirate was markedly hypercellular, with preserved trilineage haematopoiesis, reversed myeloid–erythroid ratio, and mild dyserythropoiesis (14%). Strikingly, numerous scattered foamy macrophages coexisted with characteristic blue histiocytes—displaying deeply basophilic cytoplasm on May–Grünwald Giemsa stain (Figures 1 and 2).

**FIGURE 1:**
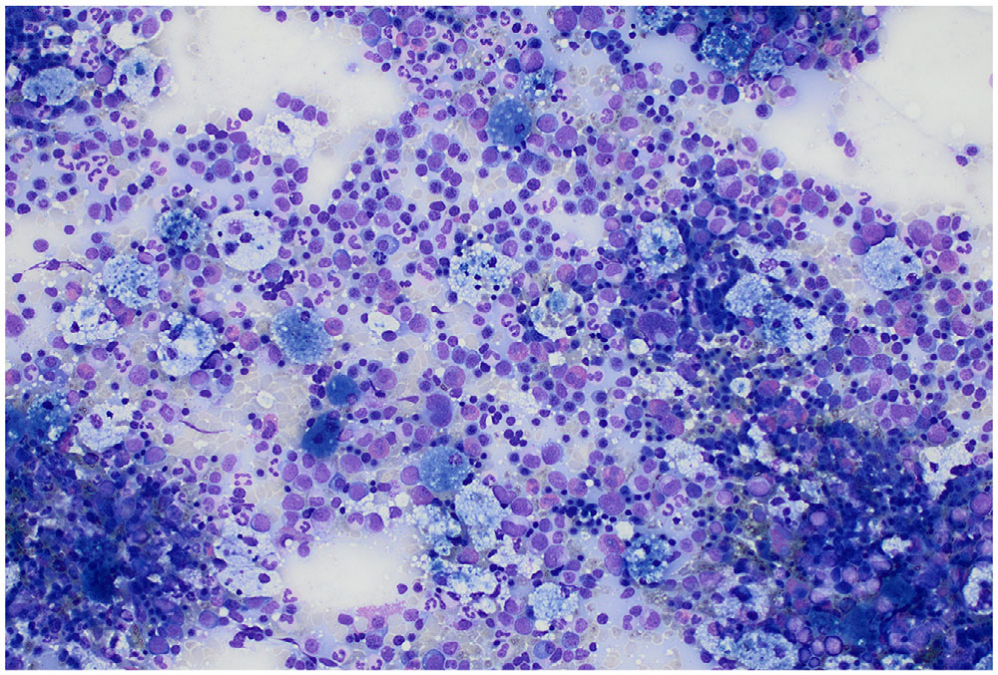
Hypercellular bone marrow aspirate (May–Grünwald Giemsa stain, ×20) showing infiltration by numerous foamy macrophages coexisting with basophilic blue histiocytes.

**FIGURE 2:**
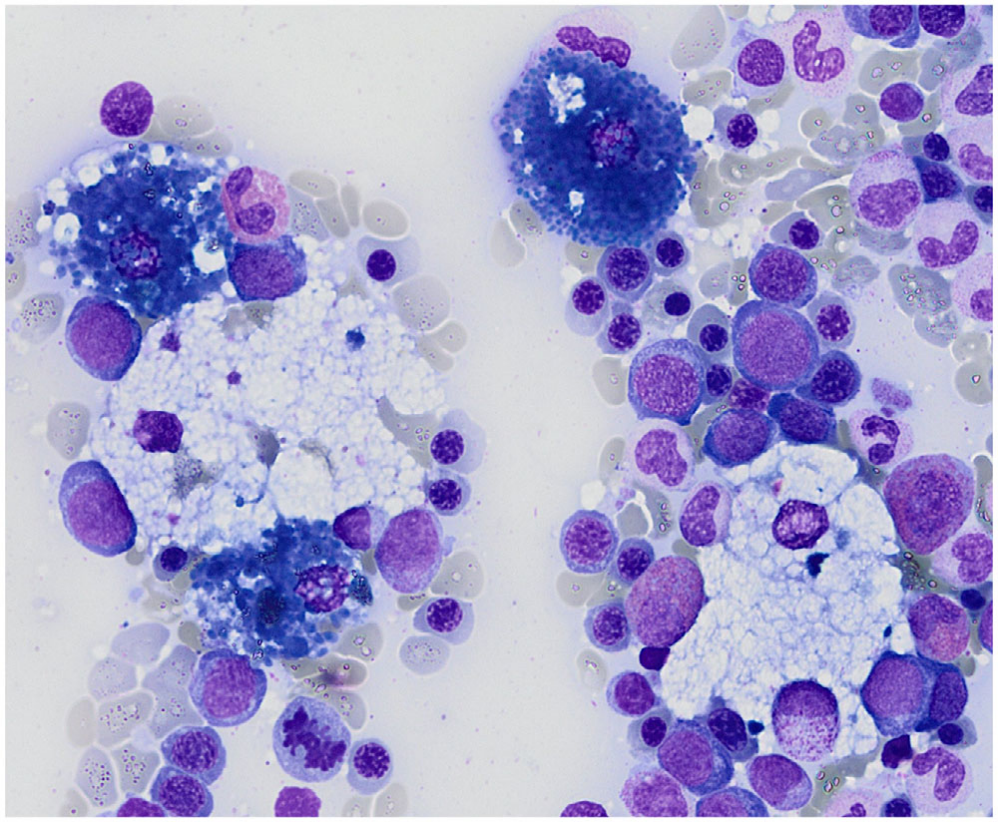
High‐power view (May–Grünwald Giemsa stain, ×100 oil immersion) highlighting foamy macrophages interspersed with intensely basophilic blue histiocytes, a cytological pattern suggestive of a lysosomal storage disorder.

Although blue histiocytes may appear as reactive findings in diverse contexts, the absence of features supporting haematologic malignancy led to targeted evaluation for inherited lysosomal storage disorders. Enzymatic testing revealed deficient acid sphingomyelinase activity, and molecular analysis confirmed acid sphingomyelinase deficiency (ASMD, formerly Niemann–Pick disease type B), due to a homozygous c.96G>A (p.Trp32*) nonsense mutation in the *SMPD1* gene, resulting in early termination of the protein. The patient initiated enzyme replacement therapy with olipudase alfa in May 2025, administered intravenously every 2 weeks with gradual dose escalation.

ASMD is a rare autosomal recessive disorder caused by lysosomal dysfunction, with organ infiltration due to lipid accumulation. Although three clinical forms are described, type B can manifest in adulthood and often evades early detection, frequently delaying diagnosis—as was the case in our patient.

This case underscores the diagnostic value of bone marrow morphology in patients with cytopenias and systemic findings. The concurrent presence of foamy macrophages and blue histiocytes should raise suspicion for lipid storage diseases, particularly ASMD, and prompt timely metabolic and genetic investigations to establish a final diagnosis and start early treatment.

## Author Contributions

All authors have contributed equally to the study.

## Ethics Statement

This manuscript respects the ethical policy of our centre for the treatment of human research participants.

## Conflicts of Interest

The authors declare no conflicts of interest.

## Data Availability

The authors have nothing to report.

